# Serum matrix metalloproteinase-13 as a diagnostic biomarker for cutaneous squamous cell carcinoma

**DOI:** 10.1186/s12885-021-08566-1

**Published:** 2021-07-15

**Authors:** Hui Wang, Hong Li, Qingtao Yan, Sumei Gao, Jianfang Gao, Zhenhua Wang, Yi Sun

**Affiliations:** 1 Department of Dermatology, Weifang People’s Hospital, 151 Guangwen St, Kuiwen District, Weifang, 261041 China; 2 Department of Pediatric Surgery, Weifang People’s Hospital, Weifang, 261041 China; 3 Department of Pathology, Weifang People’s Hospital, Weifang, 261041 China

**Keywords:** Cutaneous squamous cell carcinoma, Matrix metalloproteinase-13, Biomarker, Metastasis

## Abstract

**Background:**

A significant proportion of newly diagnosed patients with cutaneous squamous cell carcinoma (cSCC) have metastasis and eventually die of the disease, necessitating the exploration of novel biomarkers for early detection of cSCC aggressiveness, risk assessment and monitoring. Matrix metalloproteinase-13 (MMP-13) has been implicated in cSCC pathogenesis. Serum MMP-13 levels have been shown to predict survival in patients with esophageal SCC, but their diagnostic value for cSCC has not been explored.

**Methods:**

We conducted a case-control study to examine serum MMP-13 as a biomarker for cSCC. Patients with cSCC undergoing surgical resection and health controls undergoing plastic surgery were recruited. ELISA for measurement of serum MMP-13 and immunohistochemistry for detection of tissue MMP-13 were performed, and the results were compared between the case and the control group, and among different patient groups. ROC curve analysis was performed to determine the diagnostic value of serum MMP-13 levels.

**Results:**

The ratio of male to female, and the age between the case (*n* = 77) and the control group (*n* = 50) were not significantly different. Patients had significantly higher serum MMP-13 levels than healthy controls. Subjects with stage 3 cSCC had markedly higher serum MMP-13 levels than those with stage 1 and stage 2 cSCC. Patients with invasive cSCC had remarkably higher serum MMP-13 than those with cSCC in situ. Post-surgery serum MMP-13 measurement was done in 12 patients, and a significant MMP-13 decrease was observed after removal of cSCC. Tumor tissues had a remarkably higher level of MMP-13 than control tissues. Serum MMP-13 predicted the presence of invasive cSCC with an AUC of 0.87 (95% CI [0.78 to 0.95]) for sensitivity and specificity of 81.7 and 82.4%, respectively for a cut-off value of 290 pg/mL. Serum MMP-13 predicted lymph node involvement with an AUC of 0.94 (95% CI [0.88 to 0.99]) for sensitivity and specificity of 93.8 and 88.5%, respectively for a cut-off value of 430 pg/mL.

**Conclusion:**

Serum MMP-13 might serve as a valuable biomarker for early detection of cSCC invasiveness and monitoring of cSCC progression.

## Background

Matrix metalloproteinases (MMPs), a family of structurally related proteolytic enzymes, participate in the degradation of various extracellular matrix (ECM) components, e.g., collagen, elastin, fibronectin and gelatin [1, 2]. To date, over 20 MMP members have been identified in humans, which are divided into different subtypes according to their substrate specificity such as collagenase: collagenase-1 (MMP-1), collagenase-2 (MMP-8), collagenase-3 (MMP-13) and collagenase-4 (MMP-18); gelatinase: gelatinase A (MMP-2) and gelatinase B (MMP-9); and stromelysin: stromelysin-1 (MMP-3) and stromelysin-2 (MMP-10) [1]. Through regulation of ECM remodeling, MMPs play an essential role in a wide range of physiological processes, e.g., embryonic development, tissue morphogenesis, reproduction, angiogenesis, and wound healing [3–8]. Dysregulation of MMPs has been found to be involved in diverse pathological conditions including arthritis, fibrosis and neoplasia [9–23].

Cutaneous basal cell carcinoma (cBCC) and cutaneous squamous cell carcinoma (cSCC) account for approximately 80 and 20% of nonmelanoma skin cancer (NMSC), respectively [24, 25]. A systematic analysis of the global burden of disease showed that there were 7.7 million incident NMSC cases and sixty-five thousand NMSC deaths worldwide in 2017 [26]. While cBCC is a locally destructive cancer that rarely results in metastasis or death [27], cSCC is the main contributor of NMSC deaths. A number of MMPs including MMP-13 have been implicated in cSCC genesis and development [22, 28, 29]. MMP-13, also termed collagenase-3 responsible for cleavage of fibrillar collagens, gelatin and fibronectin, is not detectable in intact normal skin [30], but its expression has been shown in tumor tissues from patients with cSCC and SCC of the head and neck [30–34]. However, serum MMP-13 as a diagnostic marker for cSCC has not been explored.

## Methods

### Study subjects

Patients who had cSCC and underwent surgical procedures at our department from March 2016 to March 2019 were recruited. All methods were carried out in accordance with relevant guidelines and regulations. Diagnosis of cSCC was confirmed by pathological analysis of excised tumor tissues. Staging of cSCC was done according to the eighth edition of American Joint Committee on Cancer (AJCC) cancer staging system: T1, tumor diameter < 2 cm; T2, tumor diameter ≥ 2 cm and < 4 cm, T3, tumor diameter ≥ 4 cm, or minor bone erosion, or perineural invasion, or deep invasion; T4, tumor with gross cortical bone/bone marrow invasion [35]. Histology typing of invasive cSCC and cSCC in situ, and further subtyping of invasive cSCC into well-differentiated, moderately-differentiated and poorly-differentiated were done at our pathology department. Healthy individuals undergoing cosmetic procedures from the department of plastic surgery were recruited as controls. Patients with other skin disorders, connective tissue disease, renal disease, other tumors, hepatic disease, severe cardiovascular or pulmonary disease were excluded.

### Serum preparation

Serum samples were obtained before surgery as follows: after overnight fasting, 10 ml of whole blood from each subject was collected into serum separator tubes which were then left undisturbed at room temperature for 30 min; afterwards, blood samples were centrifuged at 2000 g for 10 min, and the resulting supernatant was collected and frozen at − 80 °C in aliquots until further analysis.

### Measurement of MMP-13 by ELISA

Serum MMP-13 was measured using the Human MMP-13 ELISA Kit obtained from Sigma China Co., Ltd. (Shanghai, China). ELISA was performed according to the manufacturer’s instructions with standards and samples run in duplicate. Briefly, 100 μl of each standard and sample were added into appropriate wells of the 96-well ELISA plate and incubated for 2.5 h at room temperature. After 4 washes with the Wash Solution, 100 μl of 1x Detection Antibody was added into each well and incubated for 1 h at room temperature followed by 4 washes with the Wash Solution. Subsequently, 100 μl of Streptavidin solution was added into each well and incubated for 45 min at room temperature followed by 4 washes as described above. Afterwards, 100 μl of TMB One-Step Substrate Reagent was added into each well and incubated for 30 min at room temperature in the dark. Finally, 50 μl of Stop Solution was added into each well, and absorbance at 450 nm of each well was read immediately using a microplate reader (BioTek, Winooski, VT, USA). Sample MMP-13 levels were determined against the concentrations of standards.

### Immunohistochemistry (IHC)

Routine tissue fixation, paraffin-embedding and sectioning, inactivation of endogenous horseradish peroxidase (HRP) and antigen retrieval were performed as described elsewhere [36]. Primary antibody incubation (1:50 dilution of anti-MMP-13 polyclonal antibodies from Boster Biological Technology, Wuhan, China) was done at room temperature for 1 h. After 3 washes with PBS, subsequent secondary antibody incubation and detection were performed using the PV-9000 IHC Kit containing biotinylated anti-rabbit secondary antibody, HRP-labeled-streptavidin, and the substrate diaminobenzidine (Zhongshan Golden Bridge Biotechnology, Beijing, China) according to the manufacturer’s instructions. Hematoxylin was used for counter staining. Under a high power field, the immune-reactive intensity was scored from 0 to 3: 0, no brown color; 1, light brown; 2, brown; and 3, dark brown. The percentage of immune-positive cells over the total cells in a high power field was scored 0–4: 0, < 5%, 1, 5–25%; 2, 26–50%; 3, 51–75% and 4, > 75%. The final immune-score was calculated by multiplying the intensity score and the percentage score, and determined as: < 3, negative; 3–5, weak; 6–8, moderate; and 9–12: strong [36]. All scorings were done by two pathologists in a blind manner and the average immune-score from 10 high power fields for each sample was compared between patients and healthy controls.

### Receiver operating characteristic (ROC) curve analysis

To determine the diagnostic value of serum MMP-13 levels for the differentiation of invasive cSCC and cSCC in situ, and the detection of cSCC lymph node metastasis, ROC curve analysis was performed using the GraphPad 8.0 statistics software (GraphPad Software Inc., San Diego, CA, USA).

### Statistical analysis

Data normality was determined by Shapiro-Wilk test. Parametric variables were presented as mean ± standard deviation and non-parametric variables as median (first quartile, third quartile). Unpaired t test for parametric variables or Mann-Whitney test for non-parametric variables was performed to analyze data between two groups of subjects. Serum MMP-13 levels among patients with different stages of cSCC and different histology subtypes (well-differentiated, moderately-differentiated and poorly-differentiated) were analyzed by one way analysis of variance (ANOVA) with post-hoc Tukey test. Categorical data were analyzed by chi-square test. *P* value < 0.05 was considered statistically significant. All statistical analyses were performed and graphs created using the GraphPad 8.0 statistics software.

## Results

A total of 77 patients (49 males and 28 females) and 50 healthy individuals (33 males and 17 females) were included in this study. For patients, fifty-seven cases of cSCC occurred in sun-exposed areas and 20 in the genital areas. The ratio of male to female, and the age in the two groups were not significantly different (Table 1).

**Table 1:** Demographic data of all participants

	Healthy controls (*n* = 50)	Patients (*n* = 77)
Gender (M/F)	33/17	49/28*
Age (years)	57.1 ± 15.9	61.3 ± 15.2^#^

* *p* = 0.79 and # *p* = 0.14 compared with controls

**Fig. 1:**
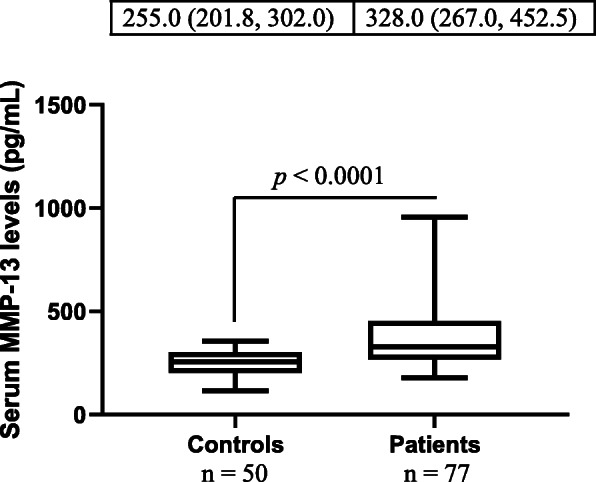
Comparison of serum MMP-13 levels between patients and healthy controls. As shown in this figure, patients with cSCC had significantly higher serum MMP-13 levels than healthy controls

**Fig. 2:**
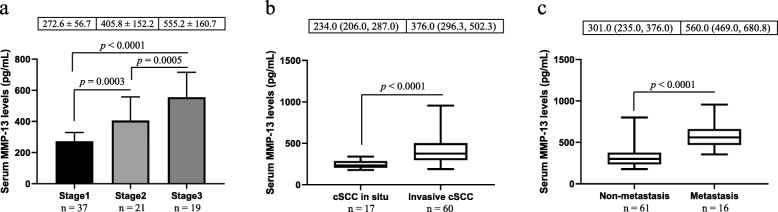
Comparison of serum MMP-13 levels among different groups of patients. Panel **a** shows the comparative results among patients with different stages of cSCC. Patients with invasive cSCC or lymph node metastasis had substantially higher serum MMP-13 levels than those with cSCC in situ (panel **b**) or without lymph node involvement (panel **c**), respectively

The cSCC group had significantly higher serum MMP-13 levels than the control group (Fig. 1). Furthermore, patients with stage 3 cSCC had markedly higher serum MMP-13 levels than those with stage 1 and stage 2 cSCC, and patients with stage 2 cSCC had substantially higher serum MMP-13 levels than individuals with stage 1 cSCC (Fig. 2 a). However, there was no significant difference in serum MMP-13 levels between healthy controls and patients with stage I cSCC. Histologically, there were 17 cases of cSCC in situ and 60 cases of invasive cSCC, and the latter had remarkably higher serum MMP-13 levels than the former (Fig. 2 b). We did not observe significant difference in serum MMP-13 levels between healthy controls and patients with cSCC in situ. Furthermore, there were no significant differences in serum MMP-13 levels among patients with well-differentiated, moderately-differentiated and poorly- differentiated cSCC (data not shown). Significantly higher levels of serum MMP-13 were also detected in subjects with lymph node metastasis compared with those without lymph node metastasis (Fig. 2 c). Patients with non-metastatic cSCC had significantly higher serum MMP-13 levels than healthy controls (*p* < 0.001). Post-surgery measurement of serum MMP-13 was performed in 12 patients, which showed a marked decrease of serum MMP-13 concentrations after the removal of cSCC (523.0 ± 231.4 pg/ml prior-surgery versus 296.4 ± 92.6 pg/ml post-surgery, p < 0.001).

**Fig. 3:**
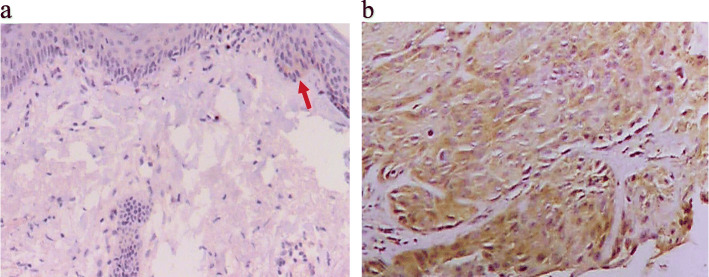
Immunohistochemical analysis of MMP-13. IHC analysis of the normal tissue from a healthy subject showed several MMP-13 positive cells (light brown staining indicated by the red arrow in panel **a**). Panel **b** is an IHC microphotograph for the cSCC tissue, and brown staining can be seen in most of tumor cells

MMP-13 expression in excised samples from all 77 patients and 30 healthy subjects were analysed by IHC. The results showed that negative and weak expression of MMP-13 were detected in 23 and 7 healthy individuals, respectively. In contrast, a significantly higher proportion of patients had positive MMP-13 expression in the resected samples (32 with moderate, 32 with weak and 13 with negative MMP-13 expression, *p* < 0.01). IHC microphotographs are shown in Fig. 3.

The age impact on serum MMP-13 levels in patients was examined. Patients were divided into two groups: one group with age <  60 years and the other with age ≥ 60 years. There were no substantial differences in serum MMP-13 levels between these two groups (Table 2). Moreover, the ratio of male to female, and the percent of patients in each stage were not markedly different between the two groups (Table 2).

**Table 2:** Comparison of serum MMP-13 levels between different age groups of patients

	< 60 years (*n* = 29)	≥ 60 years (*n* = 48)	*p* value
MMP-13 levels (pg/ml)	304.0 (252.0, 404.5)	347.5 (271.3, 484.3)	0.17
T1, n (%)	15 (51.7%)	22 (45.8%)	0.40
T2, n (%)	7 (24.1%)	14 (29.2%)	0.40
T3, n (%)	7 (24.2%)	12 (25.0%)	0.40
Gender (M/F)	20/9	29/19	0.45

MMP-13 levels were expressed as median (first quartile, third quartile). M: male; and F: female

When serum MMP-13 levels were compared between male and female patients, no significant differences were observed (Table 3). Moreover, the age and the percent of patients in each stage were not significantly different between male and female patients (Table 3).

**Table 3:** Comparison of serum MMP-13 levels between female and male patients

	Female (*n* = 28)	Male (*n* = 49)	*p* value
Serum MMP-13 levels (pg/ml)	373.5 (269.3, 518.5)	325.0 (264.0, 504.5)	0.43
T1, n (%)	13 (46.4%)	24 (49.0%)	0.97
T2, n (%)	8 (28.6%)	13 (26.5%)	0.97
T3, n (%)	7 (25.0%)	12 (24.5%)	0.97
Age (years)	61.9 ± 15.4	60.9 ± 15.3	0.78

Serum MMP-13 levels were presented as median (first quartile, third quartile)

**Fig. 4:**
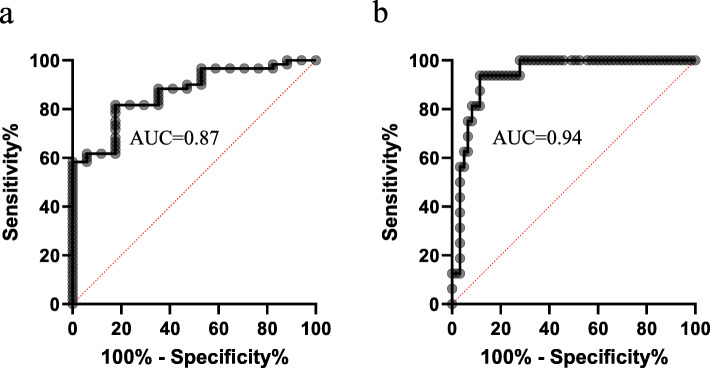
ROC curve analysis results. ROC curve analysis revealed that serum MMP-13 levels had an AUC of 0.87 and 0.94 for prediction of the presence of invasive cSCC (panel **a**) and lymph node involvement (panel **b**), respectively

The ROC curve analysis revealed that serum MMP-13 predicted the presence of invasive cSCC with an AUC of 0.87 (95% CI [0.78 to 0.95]) for sensitivity and specificity of 81.7 and 82.4%, respectively for a cut-off value of 290 pg/mL (Fig. 4 a). Serum MMP-13 predicted lymph node involvement with an AUC of 0.94 (95% CI [0.88 to 0.99]) for sensitivity and specificity of 93.8 and 88.5%, respectively for a cut-off value of 430 pg/mL (Fig. 4 b).

## Discussion

In the present study, we examined the diagnostic value of serum MMP-13 for cSCC, and reported the following findings: 1) patients with cSCC had significantly higher serum MMP-13 levels than healthy controls; 2) the stage of cSCC was associated with the concentration of serum MMP-13; 3) serum MMP-13 possesses high diagnostic value for the differentiation of invasive cSCC and cSCC in situ; 4) serum MMP-13 serves as an excellent diagnostic biomarker for cSCC lymph node metastasis; 5) post-surgery serum MMP-13 measurement was done in 12 patients, and a significant MMP-13 decrease was observed after removal of cSCC; 6) tumor tissues had a remarkably higher level of MMP-13; and 7) age and gender were not related to the elevation of serum MMP-13 levels in patients.

Although surgical resection is effective in the treatment of cSCC, it has been shown that 14% of cSCC cases at the first diagnosis have metastasis and of these patients, 40% will eventually die [37]. Currently, biomarkers that can be used for early detection of cSCC aggressiveness, risk assessment and monitoring are lacking [37–39]. Johansson et al. revealed that MMP-13 mRNA was expressed in head and neck SCC cell lines but not in normal intact skin [30]. The same group also showed that MMP-13 protein was expressed in cSCC tissues as assessed by immunohistochemistry [32]. In another study, Culhaci et al. observed a positive correlation between the degree of immunostaining for MMP-13 in tumor tissues and the invasiveness of tumor in head and neck SCC [33]. These findings suggest that analysis of MMP-13 expression in small biopsy samples may be used to determine the invasive capacity of SCC at an earlier stage. However, an objective method for quantifying MMP-13 or other molecules in SCC tissues for diagnostic purposes has not been reported. It is known that gene dysregulations caused by tumors are often reflected by the changes of final gene products in blood which can be applied for tumor diagnosis [40, 41]. In view of this and the finding of MMP-13 up-regulation in SCC tissues, we sought to explore serum MMP-13 as a diagnostic marker for cSCC, and our results show that serum MMP-13 has high sensitivity and specificity for the differentiation of invasive cSCC and cSCC in situ (Fig. 4 a), and prediction of cSCC lymph node metastasis (Fig. 4 b).

Measurement of serum MMP levels in patients with SCC of different anatomic sites, and comparison of the results with those of healthy controls have been documented [42–50]. Jiao et al. showed that patients with esophageal SCC had significantly higher serum MMP-13 levels than healthy controls; furthermore, serum MMP-13 levels were found to be associated with tumor progression and survival [42]. Riedel et al. discovered the elevation of serum MMP-9 levels in patients with head and neck SCC [43], which is also observed by Stanciu et al. [44]. Lotfi et al. reported that both serum MMP-2 and MMP-9 levels were significantly increased in laryngeal SCC cases compared with healthy controls [45], and similar results were depicted by Matulka et al. and Grzelczyk et al. [46, 47]. Choudhry et al. revealed that serum levels of MMP-1, − 8, − 10, − 12 and − 13 in oral SCC patients were substantially elevated as compared with healthy controls [48]. In contrast, Ghallab showed the increase of serum MMP-9 in oral SCC patients compared with subjects with oral premalignant lesions [49]. Among all these studies, only two groups examined the diagnostic value of MMPs: using the ROC analysis, Ghallab showed serum MMP-9 with an AUC of 0.6, which failed to differentiate between oral SCC and oral premalignant lesions [49]. Of note, serum MMP-12 was shown to have an AUC of 0.84 for sensitivity and specificity of 80 and 78.9%, respectively for a cut-off value of 16.13 pg/ml for the diagnosis of oral SCC [48]. These data together with ours suggest that serum MMPs may serve as potential biomarkers for SCC diagnosis.

We discovered that serum MMP-13 is a valuable biomarker in prediction of cSCC lymph node metastasis (Fig. 4 b). However, discrepancy in relationship between serum MMP levels and lymph node involvement has been presented [42, 44, 50]. While no significant correlations were proven between serum MMP-1, − 2, and − 9 concentrations and lymph node status in head and neck SCC [50], serum MMP-2 was found to be correlated with lymph node involvement in laryngeal SCC [45], and serum MMP-13 levels were associated with esophageal SCC lymph node metastasis [42]. These disagreeing results may be attributed to: 1) heterogeneity of SCC studied and 2) small sample sizes explored.

MMP-13 elevation in SCC cell lines or tissues has been described in several studies. Johansson et al. showed that while MMP-13 mRNA was highly expressed in head and neck SCC cell lines, it is undetectable in normal skin tissues [30]. Elevated MMP-13 mRNA expression was also observed in head and neck SCC tissues [34]. In agreement with these mRNA data, increased MMP-13 protein production in head and neck SCC tissues was detected by IHC [33]. In the present study, we found a significantly higher level of MMP-13 in cSCC tissues compared with control tissues. Moreover, serum MMP-13 substantially decreased after the resection of cSCC. These results suggest tumor-derived MMP-13 contributes to the elevation of serum MMP-13 seen in our patients.

In this study, the impact of sex and age on serum MMP-13 levels was not observed in patients, which has also been reported in SCC of different sites by other studies [42, 50]. Serum MMP-13 levels were found to be uncorrelated with age and gender in patients with esophageal SCC [42]. A study that analyzed serum levels of MMP-1, − 2, and − 9 in patients with head and neck SCC did not show correlations between serum MMP levels and sex or age [50].

## Conclusions

**S**erum MMP-13 levels show high sensitivity and specificity for the differentiation of invasive cSCC and cSCC in situ, and the prediction of lymph node metastasis, suggesting serum MMP-13 might serve as a valuable biomarker for early detection of cSCC invasiveness and monitoring of cSCC progression.

## Data Availability

The datasets supporting the conclusions of this article are included within the article.
